# A Review of Pesticide Exposure and Cancer Incidence in the Agricultural Health Study Cohort

**DOI:** 10.1289/ehp.0901731

**Published:** 2010-05-05

**Authors:** Scott Weichenthal, Connie Moase, Peter Chan

**Affiliations:** Health Canada, Ottawa, Ontario, Canada

**Keywords:** Agricultural Health Study, cancer, pesticides, review

## Abstract

**Objective:**

We reviewed epidemiologic evidence related to occupational pesticide exposures and cancer incidence in the Agricultural Health Study (AHS) cohort.

**Data sources:**

Studies were identified from the AHS publication list available at http://aghealth.nci.nih.gov as well as through a Medline/PubMed database search in March 2009. We also examined citation lists. Findings related to lifetime-days and/or intensity-weighted lifetime-days of pesticide use are the primary focus of this review, because these measures allow for the evaluation of potential exposure–response relationships.

**Data synthesis:**

We reviewed 28 studies; most of the 32 pesticides examined were not strongly associated with cancer incidence in pesticide applicators. Increased rate ratios (or odds ratios) and positive exposure–response patterns were reported for 12 pesticides currently registered in Canada and/or the United States (alachlor, aldicarb, carbaryl, chlorpyrifos, diazinon, dicamba, *S*-ethyl-*N*,*N*-dipropylthiocarbamate, imazethapyr, metolachlor, pendimethalin, permethrin, trifluralin). However, estimates of association for specific cancers were often imprecise because of small numbers of exposed cases, and clear monotonic exposure–response patterns were not always apparent. Exposure misclassification is also a concern in the AHS and may limit the analysis of exposure–response patterns. Epidemiologic evidence outside the AHS remains limited with respect to most of the observed associations, but animal toxicity data support the biological plausibility of relationships observed for alachlor, carbaryl, metolachlor, pendimethalin, permethrin, and trifluralin.

**Conclusions:**

Continued follow-up is needed to clarify associations reported to date. In particular, further evaluation of registered pesticides is warranted.

Agricultural workers are exposed to a variety of chemical, physical, and biological hazards in the process of cultivating and harvesting crops and/or raising livestock (Litchfield 1999; Popendorf and Donham 1991; Shaver and Tong 1991; White and Cessna 1989). In addition to pesticides, occupational exposure to solvents, metals, engine exhaust, welding fumes, and grain dusts are prevalent in agriculture (Coble et al. 2002; Shaver and Tong 1991). However, the potential health effects of agricultural pesticide exposures are of particular interest, as these chemicals are designed to have adverse biological effects on target organisms. To address this concern, the Agricultural Health Study (AHS) was initiated in 1993 to explore the potential health effects of pesticide exposures in commercial pesticide applicators, farmers, and their families in Iowa and North Carolina, USA. The AHS is a collaborative research project including the U.S. National Cancer Institute, the U.S. National Institute of Environmental Health Sciences, and the U.S. Environmental Protection Agency (EPA).

Details of the AHS design have been described previously (Alavanja et al. 1996). Briefly, participants were recruited between 1993 and 1997 from pesticide applicator licensing facilities in Iowa and North Carolina, and an enrollment questionnaire was used to collect data on the duration and frequency of pesticide use. In the AHS, self-reported pesticide use serves as a surrogate measure of pesticide exposure, and a cumulative pesticide exposure index (termed intensity-weighted exposure-days) is used to weigh lifetime-days (LDs) of pesticide use based on mixing conditions, application methods, and use of personal protective equipment (Dosemeci et al. 2002). Applicators who completed the enrollment questionnaire were asked to complete a take-home questionnaire that collected detailed information on factors including occupational exposures, pesticide use, lifestyle, medical history, and diet. Two additional take-home questionnaires were provided for private applicators: a spouse questionnaire and a female/family health questionnaire. Commercial applicators were asked to complete a female health questionnaire if they were female, but spouses and children of commercial applicators were not included in the AHS. A total of 52,395 private pesticide applicators (farmers or nursery workers), 32,347 spouses of private applicators, and 4,916 commercial pesticide applicators were enrolled in the AHS. Applicators are primarily male (> 95%); spouses are predominantly female (99.3%) (Alavanja et al. 2005). Private applicators, commercial applicators, and spouses are all predominantly Caucasian (94.6–98.6%) (Alavanja et al. 2005). At enrollment, 65% of private applicators reported pesticide exposure for > 11 years relative to 32% and 18% for commercial applicators and spouses of private applicators, respectively (Alavanja et al. 2005). Twelve percent of private applicators, 2.5% of commercial applicators, and 3.6% of spouses reported > 30 years of pesticide use at enrollment (Alavanja et al. 2005). Information provided at enrollment (phase 1) was updated in phase 2 (1999–2003) and phase 3 (2003–2010) of the AHS.

Approximately 80% of eligible applicators completed the enrollment questionnaire (Tarone et al. 1997). Forty percent of eligible applicators completed both the enrollment questionnaire and the take-home questionnaire (Tarone et al. 1997). Seventy-four percent of eligible spouses were enrolled in the AHS (Engel et al. 2005). Applicators who completed the take-home questionnaire tended to be older than nonrespondents but were otherwise similar to respondents with respect to pesticide use practices, medical history, and other characteristics such as education, smoking, alcohol consumption, and diet (Tarone et al. 1997). The reliability of self-reported pesticide use did not vary substantially by age, education, or farm size, and respondents generally provided plausible information regarding the duration and year of first pesticide use (Blair et al. 2002; Hoppin et al. 2002b).

In this review we focused on epidemiologic studies of pesticide exposure and cancer incidence in the AHS cohort. Studies of physical injury (Sprince et al. 2002, 2003a, 2003b, 2003c, 2007), mortality (Blair et al. 2005a, 2005b; Lee et al. 2007a), respiratory disorders (Hoppin et al. 2002a, 2006a, 2006b, 2007a, 2007b, 2008; Valcin et al. 2007), neurologic symptoms (Kamel et al. 2005, 2007a), retinal degeneration (Kamel et al. 2000; Kirrane et al. 2005), diabetes (Montgomery et al. 2008; Saldana et al. 2007), menstrual cycle characteristics (Farr et al. 2004, 2006), hearing loss (Crawford et al. 2008), Parkinson’s disease (Kamel et al. 2007b), changes in serum androgen levels (Martin et al. 2002), arthritis (De Roos et al. 2005b), depression (Beseler et al. 2006, 2008), and immune responses (Cooper et al. 2004) have also been conducted as part of the AHS but are not discussed in this review.

## Methods

We identified studies from the publication list on the AHS Web site (Agricultural Health Study 2009) and by searching the Medline/PubMed database (http://www.ncbi.nlm.nih.gov/sites/entrez?db=pubmed) using the key words “Agricultural Health Study” and “pesticides” in combination with various cancer types including “leukemia,” “prostate cancer,” “colon cancer,” and others. We also examined citation lists. Studies were included in this review if they were published before March 2009 and examined the relationship between pesticides and cancer in the AHS cohort. Findings related to LDs and/or intensity-weighted lifetime-days (IWLDs) of pesticide use are the primary focus of this review, because these measures allow for the evaluation of potential exposure–response relationships.

We identified 28 studies that examined the relationship between pesticide exposures and cancer incidence in the AHS cohort. A list of the pesticides and cancer types examined in these studies is provided in Supplemental Material (doi:10.1289/ehp.0901731). Prevalent cancer cases were excluded in all studies reviewed, and incident cases were identified by matching cohort members to state cancer registry files. Cohort members were matched to state death registries and the National Death Index to ascertain vital status, and current address records of the Internal Revenue Service, motor vehicle records, and pesticide license registries were used to identify cohort members who were alive at the end of follow-up but no longer resided in Iowa or North Carolina. Follow-up was censored at the time of cancer diagnosis, participant death, or movement out of state. In general, only a small fraction (< 5%) of study participants was lost to follow-up. Average follow-up times ranged from approximately 4 to 9 years, and when presented, standardized incidence ratios (SIRs) reflect cancer incidence in the AHS cohort relative to the populations of Iowa and North Carolina using age, sex, and race-specific incidence data. The primary pesticide exposure measures were LDs (the product of days of use per year and years of use) and IWLDs (a weighted measure of lifetime exposure-days that accounts for mixing conditions, application methods, and the use of personal protective equipment) (Dosemeci et al. 2002). Where appropriate, a number of potential confounding factors were included in Poisson/logistic regression models, including age, smoking history, alcohol consumption, education, race, sex, applicator type, state of residence, LDs of any pesticide exposure, pesticides highly correlated with the pesticide of interest, family history of cancer, year of enrollment, body mass index, sun exposure, susceptibility to sunburn, aspirin intake, physical activity, nonfarm occupational exposures, diet, menopausal status, maternal age at first birth, age at menarche, and age at menopause. Findings were reported as rate ratios (RRs) or odds ratios (ORs) and their associated 95% confidence intervals (CIs). Linear trend tests (alpha = 0.05) were used to evaluate exposure–response relationships by treating median values for each pesticide exposure category as a quantitative score or by using continuous values for LDs or IWLDs of exposure. Criteria for including a given cancer type in chemical-specific analyses (i.e., studies of one pesticide and multiple cancer types) differed between studies and ranged from a minimum total of 5 to 30 exposed cases. Alternatively, some authors specified that a given cancer type was included in chemical-specific analyses only if ≥ 4 cases were available in each category of exposure. In general, pesticides with < 5 exposed applicators were excluded from cancer-specific analyses (i.e., studies of one cancer type and multiple pesticides). Values for the median number of exposed cases included in the studies we reviewed are listed in Supplemental Material (doi:10.1289/ehp.0901731).

## Results

Study findings are summarized below for individual cancer types examined in the AHS cohort to date. Pesticides associated with cancer are listed in Table 1. A number of studies examined pesticide exposures according to both LDs and IWLDs of exposure. RRs (or ORs) for both exposure measures are included in Table 1 if at least one of these measures was associated with a significantly increased risk of cancer (*p* < 0.05). ORs were reported by Alavanja et al. (2003, 2004), Andreotti et al. (2009), and Lee et al. (2007b), and all other studies in Table 1 reported RRs. All RRs and ORs in Table 1 refer to nonexposed applicators except those reported for alachlor and all lymphohematopoietic cancers (Lee et al. 2004b), dicamba and colon (Samanic et al. 2006), and lung cancer (Alavanja et al. 2004), which refer to applicators in the lowest category of exposure.

## Study Summaries

### All cancers

Overall cancer incidence was increased among applicators in the highest exposure categories for diazinon (Beane Freeman et al. 2005) and *S*-ethyl-*N*,*N*-dipropylthiocarbamate (EPTC) (van Bemmel et al. 2008) relative to nonexposed applicators, and significant exposure–response trends were observed for both pesticides with both exposure measures. None of the other pesticides examined were associated with a significant increase in overall cancer incidence (alachlor, atrazine, captan, carbaryl, carbofuran, chlorothalonil, cyanazine, dicamba, dichlorvos, fonofos, glyphosate, heptachlor, imazethapyr, malathion, metolachlor, pendimethalin, permethrin, phorate, and trifluralin) (Bonner et al. 2005, 2007; De Roos et al. 2005a; Greenburg et al. 2008; Hou et al. 2006; Kang et al. 2008; Koutros et al. 2008, 2009; Lee et al. 2004b; Lynch et al. 2006; Mahajan et al. 2006a, 2006b, 2007; Mozzachio et al. 2008; Rusiecki et al. 2004, 2006, 2009; Samanic et al. 2006).

### Lung cancer

Applicators with the highest LDs of exposure to chlorpyrifos (Lee et al. 2004a), diazinon (Beane Freeman et al. 2005), dieldrin (Alavanja et al. 2004; Purdue et al. 2006), metolachlor (Alavanja et al. 2004), and pendimethalin (Alavanja et al. 2004; Hou et al. 2006) had increased lung cancer incidence relative to nonexposed applicators. Lung cancer risk was also significantly increased among applicators in the highest category of intensity-weighted dieldrin exposure days (Purdue et al. 2006), but diazinon and pendimethalin were not associated with lung cancer when exposures were analyzed according to this measure. Applicators with the highest LDs of dicamba exposure had increased lung cancer incidence relative to low-exposed applicators but not relative to nonexposed applicators (OR = 1.6; 95% CI, 0.7–3.4) (Alavanja et al. 2004). Significant exposure–response trends were observed for chlorpyrifos (Lee et al. 2004a), diazinon (Beane Freeman et al. 2005), dicamba (Alavanja et al. 2004), dieldrin (Alavanja et al. 2004; Purdue et al. 2006), metolachlor (Alavanja et al. 2004), and pendimethalin (Alavanja et al. 2004). However, the most recent study of pendimethalin exposure did not observe a significant exposure–response trend for lung cancer (Hou et al. 2006). Fourteen other pesticides were examined but were not associated with increased lung cancer incidence in pesticide applicators (atrazine, captan, carbaryl, chlorothalonil, cyanazine, dichlorvos, EPTC, fonofos, glyphosate, imazethapyr, malathion, permethrin, phorate, and trifluralin) (Alavanja et al. 2004; Bonner et al. 2007; De Roos et al. 2005a; Kang et al. 2008; Koutros et al. 2008, 2009; Greenburg et al. 2008; Mahajan et al. 2006a, 2006b, 2007; Mozzachio et al. 2008; Lynch et al. 2006; Rusiecki et al. 2004, 2009; Samanic et al. 2006; van Bemmel et al. 2008).

### Pancreatic cancer

Applicators in the highest categories of intensity-weighted EPTC and pendimethalin exposure-days had an increased risk of pancreatic cancer relative to nonexposed applicators, and we observed significant exposure–response trends for both pesticides (Andreotti et al. 2009). Eleven other pesticides were examined but were not associated with pancreatic cancer [alachlor, atrazine, chlorpyrifos, cyanazine, 2,4-dichlorophenoxy acetic acid (2,4-D), dicamba, glyphosate, imazethapyr, metolachlor, terbufos, and trifluralin] (Andreotti et al. 2009; De Roos et al. 2005a; Rusiecki et al. 2004).

### Colon and rectal cancer

Applicators with the highest LDs of exposure to aldicarb (Lee et al. 2007b), dicamba (Samanic et al. 2006), EPTC (van Bemmel et al. 2008), imazethapyr (Koutros et al. 2009), and trifluralin (Kang et al. 2008) had increased colon cancer incidence relative to nonexposed applicators. For imazethapyr, excess colon cancer incidence was limited to the proximal colon (Koutros et al. 2009); other studies did not examine colon cancer according to location. Significant exposure–response relationships were observed for aldicarb, dicamba, EPTC, imazethapyr, and trifluralin, but few cases were available in the first (*n* = 7) and second tertiles (*n* = 4) of intensity-weighted EPTC exposure days. Applicators with the highest lifetime exposure-days for chlordane (Purdue et al. 2006), chlorpyrifos (Lee et al. 2004a; 2007b), pendimethalin (Hou et al. 2006), and toxaphene (Lee et al. 2007b) had increased rectal cancer risk relative to nonexposed applicators. Rectal cancer risk was also significantly increased when chlorpyrifos and pendimethalin exposures were analyzed according to IWLDs (Hou et al. 2006; Lee et al. 2004a), and we observed significant exposure–response trends for chlordane (Purdue et al. 2006), chlorpyrifos (Lee et al. 2004a; 2007b), and pendimethalin (Hou et al. 2006). Seventeen other pesticides were examined but were not associated with colon or rectal cancer incidence (2,4-D, alachlor, aldrin, atrazine, captan, carbaryl, carbofuran, chlorothalonil, cyanazine, diazinon, dichlorvos, fonofos, glyphosate, malathion, metolachlor, permethrin, and phorate) (Beane Freeman et al. 2005; Bonner et al. 2005, 2007; De Roos et al. 2005a; Greenburg et al. 2008; Koutros et al. 2008; Lee et al. 2004b, 2007b; Lynch et al. 2006; Mahajan et al. 2006a, 2007; Mozzachio et al. 2008; Purdue et al. 2006; Rusiecki et al. 2004, 2006, 2009).

### All lymphohematopoietic cancers

Applicators in the highest category of intensity-weighted alachlor exposure-days had an increased incidence of all lymphohematopoietic cancers relative to low-exposed applicators (Lee et al. 2004b). Applicators in the highest categories of intensity-weighted exposure-days for chlorpyrifos (Lee et al. 2004a) and diazinon (Beane Freeman et al. 2005) had an increased incidence of all lymphohematopoietic cancers relative to nonexposed applicators, but RRs were not significantly increased when exposures were analyzed according to LDs of use. Applicators in the highest category of permethrin exposure-days had an increased incidence of all lymphohematopoietic cancers but the RR for the highest category of intensity-weighted permethrin exposure-days was not significantly increased (Rusiecki et al. 2009). Significant exposure–response trends were observed for alachlor (Lee et al. 2004b) and diazinon (Beane Freeman et al. 2005). Fifteen other pesticides were examined but were not associated with lymphohematopoietic cancers (captan, carbaryl, carbofuran, cyanazine, dicamba, dichlorvos, EPTC, fonofos, glyphosate, imazethapyr, malathion, metolachlor, pendimethalin, phorate, and trifluralin) (Bonner et al. 2005, 2007; De Roos et al. 2005a; Greenburg et al. 2008; Hou et al. 2006; Kang et al. 2008; Koutros et al. 2008, 2009; Lee et al. 2004b; Lynch et al. 2006; Mahajan et al. 2006a, 2006b, 2007; Rusiecki et al. 2006; Samanic et al. 2006).

### Leukemia

Applicators with the highest LDs of exposure for heptachlor/chlordane (Purdue et al. 2006), diazinon Beane Freeman et al. 2005, and EPTC (van Bemmel et al. 2008) had increased leukemia incidence relative to nonexposed applicators. When exposures were analyzed according to intensity-weighted exposures-days, applicators in the highest categories of exposure for fonofos (Mahajan et al. 2006a) and chlorpyrifos (Lee et al. 2004a) also had increased leukemia incidence relative to nonexposed applicators. Significant dose–response relationships were observed for heptachlor/chlordane, diazinon, EPTC, and fonofos. Seven other pesticides were examined but were not associated with leukemia in pesticide applicators (alachlor, atrazine, carbaryl, glyphosate, imazethapyr, malathion, and trifluralin) (Bonner et al. 2007; De Roos et al. 2005a; Kang et al. 2008; Koutros et al. 2009; Mahajan et al. 2007; Rusiecki et al. 2004, 2009).

### Non-Hodgkin lymphoma

Applicators in the highest category of intensity-weighted exposure-days for lindane had increased non-Hodgkin lymphoma (NHL) incidence relative to nonexposed applicators, and a significant exposure–response trend was observed (Purdue et al. 2006). However, we did not observe a significant trend for lindane when we analyzed exposures according to lifetime exposure-days. Sixteen other pesticides were examined but were not associated with NHL in pesticide applicators (alachlor, atrazine, carbaryl, carbofuran, chlorpyrifos, cyanazine, diazinon, dicamba, EPTC, glyphosate, imazethapyr, malathion, metolachlor, pendimethalin, permethrin, and trifluralin) (Beane Freeman et al. 2005; Bonner et al. 2005, 2007; De Roos et al. 2005a; Hou et al. 2006; Kang et al. 2008; Koutros et al. 2009; Lee et al. 2004a, 2004b; Lynch et al. 2006; Mahajan et al. 2007; Rusiecki et al. 2004, 2006, 2009; Samanic et al. 2006; van Bemmel et al. 2008).

### Multiple myeloma

Applicators in the highest categories of permethrin exposure had an increased incidence of multiple myeloma relative to nonexposed applicators, and we observed significant exposure–response patterns (Rusiecki et al. 2009). However, ≤ 3 cases were available in the first and second tertiles of exposure; further evaluation of a potential exposure–response pattern is required once more cases have accrued. Four other pesticides were examined but were not associated with multiple myeloma (alachlor, atrazine, chlorpyrifos, and glyphosate) (De Roos et al. 2005a; Lee et al. 2004a, 2004b; Rusiecki et al. 2004).

### Breast cancer

Engel et al. (2005) examined breast cancer incidence among farmers’ wives. Breast cancer incidence was decreased among women who reported ever applying pesticides relative to the general population (SIR = 0.87; 95% CI, 0.89–1.24), and strong associations were not detected for specific pesticides. Ever use of pesticides in this study included use on crops and livestock as well as use in the home or garden. Although few women personally applied many of the pesticides examined, breast cancer incidence was increased among women whose husbands reported ever use of aldrin (RR = 1.9; 95% CI, 1.3–2.7), carbaryl (RR = 1.4; 95% CI, 1.0–2.0), chlordane (RR = 1.7; 95% CI, 1.2–5.5), dieldrin (RR = 2.0; 95% CI, 1.1–3.3), heptachlor (RR = 1.6; 95% CI, 1.1–2.4), lindane (RR = 1.7; 95% CI, 1.1–2.5), malathion (RR = 1.4; 95% CI, 1.0–2.0), 2,4,5-trichlorophenoxypropionic acid (2,4,5-TP) (RR = 2.0; 95% CI, 1.2–3.2) or captan (RR = 2.7; 95% CI, 1.7–4.3). RRs varied by menopausal status, with increased breast cancer incidence observed among premenopausal women ever exposed to chlorpyrifos (RR = 2.2; 95% CI, 1.0– 4.9), dichlorvos (RR = 2.3; 95% CI, 1.0–5.3), or terbufos (RR = 2.6; 95% CI, 1.1–5.9) but not among postmenopausal women. Potential exposure–response patterns were not examined in this study because pesticide exposure information was limited to ever/never use data. Other studies of pesticide exposure and breast cancer incidence in the AHS cohort have not been conducted to date.

### Bladder cancer

Applicators in the highest category of intensity-weighted imazethapyr exposure-days had an increased incidence of bladder cancer relative to nonexposed applicators, and a significant exposure–response pattern was observed (Koutros et al. 2009). Nine other pesticides were examined but were not associated with bladder cancer in pesticide applicators (alachlor, atrazine, carbaryl, dicamba, EPTC, glyphosate, malathion, permethrin, and trifluralin) (Bonner et al. 2007; De Roos et al. 2005a; Kang et al. 2008; Lee et al. 2004b; Mahajan et al. 2007; Rusiecki et al. 2004, 2009; Samanic et al. 2006; van Bemmel et al. 2008).

### Prostate cancer

Applicators in the highest categories of fonofos exposure who also had a family history of prostate cancer had increased prostate cancer incidence relative to nonexposed applicators (Mahajan et al. 2006a). Significant exposure–response patterns were observed for both exposure measures, and the reported findings suggest that a family history of prostate cancer may modify prostate cancer risk in applicators exposed to fonofos. Applicators in the highest categories of fonofos exposure without a family history of prostate cancer did not have increased prostate cancer incidence (RR_LD_ = 0.86; 95% CI, 0.60–1.24; RR_IWLD_ = 0.96; 95% CI, 0.70–1.31). Applicators in the highest category of intensity-weighted exposure-days for methyl bromide had increased prostate cancer risk relative to nonexposed applicators, and a significant exposure–response trend was observed (Alavanja et al. 2003). Twenty-three other pesticides were examined but were not associated with prostate cancer (alachlor, aldrin, atrazine, captan, carbofuran, carbaryl, chlorothalonil, chlorpyrifos, cyanazine, DDT, diazinon, dicamba, dichlorvos, EPTC, glyphosate, heptachlor, imazethapyr, malathion, metolachlor, pendimethalin, permethrin, phorate, and trifluralin) (Alavanja et al. 2003; Beane Freeman et al. 2005; Bonner et al. 2005, 2007; De Roos et al. 2005a; Greenburg et al. 2008; Hou et al. 2006; Kang et al. 2008; Koutros et al. 2008, 2009; Lynch et al. 2006; Mahajan et al. 2006b, 2007; Mozzachio et al. 2008; Rusiecki et al. 2004, 2006, 2009; Samanic et al. 2006; van Bemmel et al. 2008).

### Brain cancer

Lee et al. (2004a) examined brain cancer incidence in pesticide applicators exposed to chlorpyrifos. Applicators in the highest category of intensity-weighted chlorpyrifos exposure-days had increased brain cancer incidence relative to nonexposed applicators, and a significant exposure–response pattern was observed. However, findings were based on small numbers of exposed cases (2 ≤ *n* ≤ 7) and the exposure–response trend was not monotonic, as the second-highest exposure group had a lower risk of brain cancer (RR = 1.25; 95% CI, 0.26–6.10) than applicators in the lowest category of exposure (RR = 3.32; 95% CI, 0.98–11.24). Elevated RRs were reported for the two highest categories of chlorpyrifos exposure-days, but these estimates were not significantly increased and a significant exposure–response trend was not observed. Other studies of pesticide exposure and brain cancer incidence in the AHS cohort have not been conducted to date.

### Melanoma

Applicators in the highest categories of lifetime carbaryl (Mahajan et al. 2007) and toxaphene (Purdue et al. 2006) exposure-days had an increased incidence of melanoma relative to nonexposed applicators. Significant exposure–response patterns were not observed for carbaryl when exposures were analyzed according to lifetime exposure-days or intensity-weighted exposure-days (Mahajan et al. 2007). We observed a significant exposure–response trend with increasing LDs of toxaphene exposure but not when exposures were analyzed according to intensity-weighted exposure-days. Ten other pesticides were examined but were not associated with melanoma incidence in pesticide applicators (atrazine, diazinon, dicamba, EPTC, fonofos, glyphosate, imazethapyr, malathion, pendimethalin, and permethrin) (Beane Freeman et al. 2005; Bonner et al. 2007; De Roos et al. 2005a; Hou et al. 2006; Koutros et al. 2009; Mahajan et al. 2006a; Rusiecki et al. 2004, 2009; Samanic et al. 2006; van Bemmel et al. 2008).

### Other cancers

#### Kidney cancer

Six studies examined the relationship between pesticide exposure and kidney cancer in pesticide applicators, but strong associations were not observed for any of the pesticides examined (atrazine, chlorpyrifos, glyphosate, imazethapyr, malathion, and trifluralin) (Bonner et al. 2007; De Roos et al. 2005a; Kang et al. 2008; Koutros et al. 2009; Lee et al. 2004a; Rusiecki et al. 2004). Increased RRs were reported for applicators in the highest categories of trifluralin exposure relative to nonexposed applicators (RR_LD_ = 2.06; 95% CI, 0.75–5.65; RR_IWLD_ = 1.77; 95% CI, 0.73–4.30), but these estimates were not significantly increased, and significant exposure–response trends were not observed for either exposure measure (Kang et al. 2008).

#### Childhood cancer

Flower et al. (2004) examined cancer incidence in children of male farmers in Iowa. Overall cancer incidence was increased among children of pesticide applicators (SIR = 1.36; 95% CI, 1.03–1.79), and more lymphoma (SIR = 2.18; 95% CI, 1.13– 4.19) and Hodgkin lymphoma (SIR = 2.56; 95% CI, 1.06–6.14) cases were observed than expected based on childhood cancer rates in the Iowa population. However, SIRs for specific cancers were based on small numbers of cases (2 ≤ *n* ≤ 11). Cancer risk was increased among children whose fathers did not use chemically resistant gloves when mixing pesticides relative to children with fathers who wore gloves (OR = 1.98; 95% CI, 1.05–3.76). In addition, relative to children of nonexposed men, children whose fathers used aldrin during the prenatal period also had an increased cancer risk (OR = 2.66; 95% CI, 1.08–6.59). Potential exposure–response patterns were not examined for specific pesticides, however, and 15 other pesticides examined in this study were not associated with childhood cancer (alachlor, atrazine, chlorpyrifos, cyanazine, 2,4-D, dichlorvos, dicamba, EPTC, glyphosate, malathion, metolachlor, metribuzin, phorate, trifluralin, and terbufos).

#### Miscellaneous

Several studies examined the relationship between pesticide exposures and oral cavity cancers (De Roos et al. 2005a; Koutros et al. 2009; Rusiecki et al. 2004, 2006), stomach cancer (Lee et al. 2004b), esophagus cancer (Lee et al. 2004a; Rusiecki et al. 2004), and thyroid cancer (Lee et al. 2004b), but none of the six pesticides examined were associated with increased risk of these types of cancers (alachlor, atrazine, chlorpyrifos, glyphosate, imazethapyr, and metolachlor).

## Discussion

Through March 2009, 27 studies examined the relationship between LDs or IWLDs of pesticide exposure and cancer incidence in the AHS cohort. Thirty-two different pesticides were included in these studies, and most study participants personally applied pesticides for 11– 30 years before enrollment (Alavanja et al. 2005). When appropriate, all studies adjusted for the use of pesticides correlated with the specific pesticide of interest; however, it is possible that this adjustment did not completely isolate the independent effects of each individual pesticide or eliminate the impact of multiple exposures. Nonetheless, findings from Coble et al. (2002) suggest that the magnitude of bias due to confounding from exposure to multiple agents is likely to be minimal based on the proportion of farmers reporting exposure to agents including cleaning solvents and diesel exhaust. Findings of chemical cohort analyses (i.e., studies of a single pesticide and multiple cancer types) and cancer site analyses (i.e., studies of a single cancer type and multiple pesticide exposures) were generally consistent with respect to the magnitude and direction of the observed associations. Specifically, chemical cohort and cancer site analysis were consistent for carbaryl and colon cancer (no association) (Lee et al. 2007b; Mahajan et al. 2007), chlorpyrifos and rectal cancer (significantly increased risk) (Lee et al. 2004a, 2007b), pendimethalin, permethrin, and colorectal cancer (no association) (Hou et al. 2006; Lee et al. 2007b; Rusiecki et al. 2009), carbofuran, chlorpyrifos, diazinon, dieldrin, pendimethalin, and lung cancer (significantly increased risk) (Alavanja et al. 2004; Beane Freeman et al. 2005; Bonner et al. 2005; Hou et al. 2006; Lee et al. 2004a; Purdue et al. 2006), dicamba and lung cancer (no association with nonexposed reference group) (Alavanja et al. 2004; Samanic et al. 2006), atrazine, glyphosate, and pancreatic cancer (no association) (Andreotti et al. 2009; De Roos et al. 2005a; Rusiecki et al. 2004), and atrazine, captan, carbofuran, permethrin, and prostate cancer (no association) (Alavanja et al. 2003; Bonner et al. 2005; Greenburg et al. 2008; Rusiecki et al. 2004, 2009). Findings of Lee et al. (2007b) and Purdue et al. (2006) were somewhat inconsistent with respect to the relationship between chlordane and rectal cancer. Specifically, Purdue et al. (2006) reported an increased RR for applicators with > 9 LDs of chlordane exposure, whereas Lee et al. (2007b) did not observe increased rectal cancer incidence among applicators with > 56 days of exposure. However, in the analysis conducted by Lee et al. (2007b), only two exposed cases were available in the highest exposure group (> 56 LDs), and rectal cancer incidence was increased among applicators with 20–56 days of chlordane exposure. Therefore, both studies provide evidence of an association between chlordane and rectal cancer even though Lee et al. (2007b) did not observe an increased OR in the highest category of exposure. Chemical cohort analysis for metolachlor (Rusiecki et al. 2006) did not confirm the previously observed association with lung cancer (Alavanja et al. 2004). One explanation for this discrepancy may be that the association reported by Alavanja et al. (2004) occurred by chance, as findings were based on fewer exposed cases and were less precise. Alternatively, differences in cutoff points used for the highest exposure groups may explain this inconsistency; a value of 457 LDs was used by Alavanja et al. (2003), whereas a value of 116 LDs was used by Rusiecki et al. (2006). Therefore, it is possible that an increased RR was not reported by Rusiecki et al. (2006) because the highest exposure group included applicators with exposure levels below those likely to result in increased cancer risk. This possibility is supported by the fact that Alavanja et al. (2003) did not observe an association between metolachlor and lung cancer among applicators exposed for 116–457 LDs. Finally, increased risks of colon cancer observed in chemical specific analyses for dicamba (Samanic et al. 2006) and trifluralin (Kang et al. 2008) do not agree with findings reported in cancer-specific analyses (Lee et al. 2007b). However, analyses for dicamba and trifluralin were limited to ever/never exposure classification in the study by Lee et al. (2007b), whereas Samanic et al. (2006) and Kang et al. (2008) reported increased RRs for colon cancer when exposures were analyzed according to intensity-weighted exposure days. Therefore, differences in exposure classification may account for discrepancies observed between these studies.

In total, 19 pesticides were associated with a significantly increased risk of at least one type of cancer. Clear similarities by type (i.e., insecticide, herbicide, or fungicide), chemical structure, or chemical family were not apparent among these pesticides (Table 1). Seven of these 19 pesticides are no longer registered for use in Canada or the United States (chlordane, dieldrin, fonofos, heptachlor, lindane, methyl bromide, and toxaphene), and three additional pesticides, alachlor, aldicarb, and metolachlor, are registered for use in the United States but are not registered in Canada. Of the remaining pesticides currently registered for use in Canada or the United States, statistically significant exposure–response trends were observed for alachlor (all lymphohematopoietic), aldicarb (colon), carbaryl (melanoma), chlorpyrifos (lung, rectal), diazinon (all cancers, all lymphohematopoietic, leukemia, lung), dicamba (colon, lung), EPTC (all cancers, colon, pancreas), imazethapyr (bladder, colon), metolachlor (lung), pendimethalin (lung, pancreas, rectal), permethrin (multiple myeloma), and trifluralin (colon) (Alavanja et al. 2004; Andreotti et al. 2009; Beane Freeman et al. 2005; Hou et al. 2006; Kang et al. 2008; Koutros et al. 2009; Lee et al. 2004a, 2004b, 2007b; Mahajan et al. 2007; Rusiecki et al. 2006, 2009; Samanic et al. 2006; van Bemmel et al. 2008). These pesticides are listed in Table 2 along with animal toxicologic evidence of carcinogenicity noted by the U.S. EPA (2007), Canadian Pest Management Regulatory Agency (PMRA) (Health Canada 2003, 2005, 2007, 2008), and the International Agency for Research on Cancer (IARC 2010). The U.S. EPA classification terms “likely” and “not likely” in Table 2 do not correspond to quantifiable probabilities of carcinogenicity but instead reflect the weight of animal toxicologic evidence for or against such a relationship (i.e., a classification of “likely” does not mean that a given pesticide is a confirmed carcinogen, but only that such an effect is plausible give current toxicologic evidence).

The IARC has not evaluated most pesticides listed in Table 2 (alachlor, chlorpyrifos, diazinon, dicamba, EPTC, imazethapyr, metolachlor, pendimethalin, and trifluralin) and considers the remaining pesticides (aldicarb, carbaryl, and permethrin) not classifiable with respect to human carcinogenicity (group 3). Evidence of carcinogenicity was noted by the U.S. EPA and/or PMRA in animal toxicity studies for alachlor, carbaryl, metolachlor, pendimethalin, permethrin, and trifluralin, thus supporting the biological plausibility of associations observed for these pesticides. The remaining registered pesticides for which exposure–response relationships were observed are not considered carcinogenic by the U.S. EPA or PMRA (aldicarb, chlorpyrifos, diazinon, dicamba, EPTC, and imazethapyr).

Risk estimates were imprecise for most registered pesticides that displayed an exposure–response pattern with at least one type of cancer because of small numbers of exposed cases. Specifically, ≤ 12 cases were available in the highest categories of exposure for aldicarb (colon), carbaryl (melanoma), chlorpyrifos (rectal), diazinon (all LH, leukemia, lung), dicamba (lung), EPTC (pancreas), imazethapyr (colon, bladder), metolachlor (lung), pendimethalin (lung, pancreas, rectal), and permethrin (multiple myeloma). As a result, little can be concluded at this time regarding the causal nature of these associations, and further analyses are required once more cases have accrued. RRs for chlorpyrifos and lung cancer and EPTC and colon cancer were more precise, each indicating that cancer incidence doubled in the highest exposure groups relative to nonexposed applicators (Lee et al. 2004a; van Bemmel et al. 2008). However, the weight of biological evidence reviewed by the U.S. EPA and PMRA does not suggest that chlorpyrifos and EPTC are carcinogenic (Health Canada 2003, 2008; Smegal 2002; U.S. EPA 1999). One explanation for discrepancies between AHS findings and toxicologic evidence may be that animal toxicity tests typically reflect exposure to a single pesticide active ingredient and not the end-use product or multiple products. Therefore, real-life exposures in the field are not equivalent to what is tested in animal toxicity studies. Alternatively, some AHS findings may have occurred by chance because of the large number of multiple comparisons or bias from uncontrolled confounding or other source of bias. Nevertheless, as many of the studies reviewed are the first to examine the reported associations, the findings are useful for generating hypotheses that require confirmation in future studies. Going forward, adjustment for multiple comparisons will be important to avoid spurious associations. Likewise, bias analysis may be helpful in characterizing overall uncertainty in AHS findings; a recent study by Lash (2007) illustrates how conventional frequentist methods may understate uncertainty in effect measures by quantifying only random error and may result in bias away from the null.

Epidemiologic evidence outside the AHS cohort remains limited with respect to associations observed for specific pesticides and cancer types listed in Table 2. Three studies examined cancer incidence in a cohort of alachlor manufacturing workers in Iowa (Acquavella et al. 1996, 2004; Leet et al. 1996). Lymphohematopoietic tumors were increased in one of these studies relative to expected values in the Iowa population (SIR = 3.6; 95% CI, 1.2–8.5) (Leet et al. 1996); however, this estimate was based on only five exposed cases, and potential confounding factors were not included in the analyses. Two or fewer lymphohematopoietic cancer cases were available in studies conducted by Acquavella et al. (1996, 2004), and in general, none of the three studies of alachlor manufacturing workers provides strong evidence of an important relationship between alachlor and cancer. Studies of carbaryl and melanoma have not been conducted outside the AHS cohort, but Zheng et al. (2001) reported an association between small-cell lymphoma among participants exposed to carbaryl in a pooled analysis of three case–control studies in the United States (Cantor et al. 1992; Hoar et al. 1986; Zahm et al. 1990). However, carbaryl exposure was not associated with NHL in a Canadian case–control study after adjusting for a number of potential confounding factors (McDuffie et al. 2001), and carbaryl was not associated with NHL in the AHS cohort (Mahajan et al. 2007). A case–control study examined lung cancer mortality in Florida pest control workers exposed to chlorpyrifos and diazinon, but neither pesticide was associated with a significantly increased risk of lung cancer mortality (Pesatori et al. 1994). Chlorpyrifos and diazinon were identified as possible risk factors for NHL in a pooled analysis of three case–control studies in Iowa, Minnesota, Kansas, and Nebraska (Waddell et al. 2001); however, these pesticides were not associated with NHL in the AHS cohort (Beane Freeman et al. 2005; Lee et al. 2004a). One previous case–control study observed an association between dicamba and NHL (McDuffie et al. 2001), but dicamba was not associated with NHL in the AHS cohort. Hoar et al. (1986) noted an association between NHL and trifluralin exposure based on only three exposed cases, but a more recent pooled analysis did not observe a significant relationship between trifluralin and NHL (De Roos et al. 2003). Previous studies of chlorpyrifos and rectal cancer and diazinon and leukemia were not identified. Likewise, studies of aldicarb, dicamba, and colon cancer, EPTC and colon or pancreatic cancer, imazethapyr and bladder or colon cancer, metolachlor and lung cancer, pendimethalin and lung, rectal, or pancreatic cancer, permethrin and myeloma, and trifluralin and colon cancer were not identified outside the AHS cohort, and epidemiologic evidence in general is limited for these pesticides.

Exposure assessment is a challenge in large-scale epidemiologic studies, as it is often not possible to obtain quantitative exposure data at etiologically relevant time periods for large numbers of study participants. In the AHS, self-reported LDs and IWLDs of pesticide exposure are used as the primary exposure measures. To date, four studies have examined the validity of the intensity-weighted exposure algorithm used in the AHS (Acquavella et al. 2006; Coble et al. 2005; Thomas et al. 2009). Two of these studies were conducted using data from the Pesticide Exposure Assessment Study conducted in Canada (Acquavella et al. 2006; Coble et al. 2005), and the remaining two studies were conducted using members of the AHS cohort (Hines et al. 2008; Thomas et al. 2009). In general, validation studies have observed low to moderate correlations between exposure intensity algorithm scores and urinary biomarkers of 2,4-D, 4-chloro-2-methylphenoxyacetic acid (MCPA), captan, glyphosate, and chlorpyrifos (Acquavella et al. 2006; Coble et al. 2005; Hines et al. 2008; Thomas et al. 2009). Low to moderate correlations were also reported between algorithm scores and quantitative levels of 2,4-D and chlorpyrifos measured in hand-wipe samples, dermal patches, and personal air samples (Thomas et al. 2009); however, correlations between algorithm scores and quantitative measures of chlorpyrifos exposure varied by application method, with stronger correlations observed for liquid spray applications relative to granular in-furrow applications. For captan, intensity-weighted algorithm scores were predictive of exposure levels measured on dermal patch samples located on the thighs of pesticide applicators but were not significant predictors of captan levels measured in personal air samples, hand rinses, and forearm patches (Hines et al. 2008). Weighted kappa values for categorical agreement between algorithm scores and biomarker levels were low to moderate (0.07 < kappa < 0.37) in validation studies conducted to date, and considerable overlap in urinary biomarker levels was apparent between exposure categories based on algorithm scores (Acquavella et al. 2006; Thomas et al. 2009). However, algorithm scores were able to detect significant trends in urine and hand-wipe concentrations of 2,4-D (Coble et al. 2005; Thomas et al. 2009) and urine concentrations of MCPA (Coble et al. 2005), and in general, biomarker levels tended to be greatest among participants labeled as having the highest exposures (Acquavella et al. 2006; Thomas et al. 2009).

Exposure misclassification undoubtedly had an impact on AHS findings reported to date. As participants reported exposures prior to disease onset, the process of exposure misclassification in the AHS cohort is likely to be nondifferential; however, this does not guarantee bias toward the null in any individual study (Dosemeci et al. 1990; Jurek et al. 2005; 2008; Pearce et al. 2007; Sorahan and Gilthorpe 1994; Thomas 1995). What seems apparent from validation studies is that the exposure intensity algorithm is capable of differentiating subjects with the highest and lowest exposure levels but is less capable of valid exposure classification across an exposure gradient. Unfortunately, this limits the ability to detect exposure–response patterns, as substantial exposure misclassification is expected to occur across categories of exposure. Further validation of the exposure intensity algorithm in biomonitoring studies for an expanded group of pesticides may help to characterize this uncertainty. Similarly, the applicability of intensity-weighted exposure measures in studies of lung cancer requires further evaluation, as the algorithm weighs dermal exposures most heavily (Dosemeci et al. 2002) and may not offer an improvement over lifetime-exposure days for outcomes related to inhalation exposures. Finally, it is not clear how current exposure levels compare with those during etiologically relevant time periods, as the first years of pesticide use often occurred decades prior to enrollment in the AHS.

## Conclusions

We reviewed 28 studies of pesticide exposure and cancer incidence in the AHS cohort. Most of the 32 pesticides examined were not strongly associated with cancer, but increased RRs/ORs and positive exposure–response relationships were observed for 12 pesticides currently registered in Canada and/or the United States. However, RRs and ORs were often imprecise because of small numbers of exposed cases, and further follow-up is required once more cases have accrued. Epidemiologic evidence outside the AHS cohort remains limited with respect to most of the observed associations, but animal toxicity data support the possible carcinogenicity of alachlor, carbaryl, metolachlor, pendimethalin, permethrin, and trifluralin. Although the exposure intensity algorithm developed for the AHS offers an improvement over ever/never exposure classification often employed in environmental health studies, exposure misclassification remains a concern. In particular, analysis of exposure–response trends is limited by expected exposure misclassification across categories of LDs and IWLDs. Further validation of the exposure intensity algorithm for an expanded group of pesticides will help to characterize uncertainty resulting from exposure misclassification. In addition, continued follow-up of the AHS cohort as a whole will help to clarify associations reported to date. In doing so, particular attention should be paid to registered pesticides that displayed evidence of a possible association with cancer.

## Figures and Tables

**Table 1:** Pesticides associated with cancer in the AHS cohort.

Cancer type	Pesticide(s)	Chemical family	Categorical exposure cutoff value	RR or OR^a^ (95% CI)	*p*-Value for trend	References
All cancers	Diazinon	OP	> 109 LD^b^	1.58 (1.10–2.28)	0.007	Beane Freeman et al. 2005
			Highest IWLD^b^	1.41 (1.03–1.95)	0.033	

	EPTC	Thiocarbamate	> 50 LD^c^	1.28 (1.09–1.50)	< 0.01	van Bemmel et al. 2008
			> 112 IWLD^c^	1.16 (1.01–1.35)	0.02	

Lung	Chlorpyrifos	OP	> 56 LD^b^	2.18 (1.31–3.64)	0.002	Lee et al. 2004a
			> 417 IWLD^b^	1.80 (1.00–3.23)	0.036	

	Diazinon	OP	> 109 LD^b^	3.46 (1.57–7.65)	0.001	Beane Freeman et al. 2005
			Highest IWLD^b^	1.55 (0.65–3.72)	0.22	

	Dicamba	Benzoic acid	> 224 LD^b^	3.10 (1.20–7.70)	0.04	Alavanja et al. 2004

	Dieldrin	OC	> 50 LD^b^	5.30 (1.50–18.6)	0.005	
			> 9 LD^d^	2.80 (1.10–7.20)	0.02	Purdue et al. 2006

	Metolachlor	Chloroacetanilide	Highest IWLD^d^	3.50 (1.60–7.70)	0.002	
			> 457 LD^b^	4.10 (1.60–10.4)	0.015	Alavanja et al. 2004

	Pendimethalin	Dinitroaniline	> 224 LD^b^	3.50 (1.10–10.5)	0.005	
			>116 LD^b^	2.40 (1.10–5.30)	0.29	Hou et al. 2006
			> 539 IWLD^b^	1.10 (0.50–2.60)	0.94	

Pancreas	EPTC	Thiocarbamate	> 118 IWLD^d^	2.50 (1.10–5.40)	0.01	Andreotti et al. 2009

	Pendimethalin	Dinitroaniline	> 117 IWLD^d^	3.00 (1.30–7.20)	0.01	

Colon	Aldicarb	Carbamate	> 56 LD^b^	4.10 (1.30–12.8)	0.001	Lee et al. 2007a

	Dicamba	Benzoic acid	> 116 LD^b^	3.29 (1.40–7.73)	0.02	Samanic et al. 2006
			> 739 IWLD^b^	2.57 (1.28–5.17)	0.002	

	EPTC	Thiocarbamate	> 50 LD^c^	2.09 (1.26–3.47)	< 0.01	van Bemmel et al. 2008
			> 112 IWLD^c^	2.05 (1.34–3.14)	< 0.01	

	Imazethapyr	Imidazolinone	> 311 IWLD (proximal)^b^	2.73 (1.42–5.25)	0.001	Koutros et al. 2009
			> 311 IWLD (distal)^b^	1.21 (0.55–2.68)	0.75	

	Trifluralin	Dinitroaniline	> 224 LD^b^	1.48 (0.78–2.80)	0.12	Kang et al. 2008
			> 1176 IWLD^b^	1.76 (1.05–2.95)	0.036	

Rectum	Chlordane	OC	> 9 LD^d^	2.70 (1.10–6.80)	0.03	Purdue et al. 2006
			Highest IWLD^d^	2.10 (0.90–5.30)	0.04	

	Chlorpyrifos	OP	> 56 LD^b^	3.25 (1.60–6.62)	0.035	Lee et al. 2004a
			> 417 IWLD^b^	3.16 (1.42–7.03)	0.057	
			>109 LD^b^	2.70 (1.20–6.40)	0.008	Lee et al. 2007a

	Pendimethalin	Dinitroaniline	> 116 LD^c^	4.30 (1.50–12.7)	0.007	Hou et al. 2006
			> 539 IWLD^c^	3.60 (1.20–11.3)	0.02	

	Toxaphene	OC	> 56 LD^b^	4.30 (1.20–15.8)	0.123	Lee et al. 2007a

Leukemia	Chlordane/Heptachlor	OC	> 9 LD^d^	2.60 (1.20–6.00)	0.02	Purdue et al. 2006
			Highest IWLD^d^	2.10 (0.80–5.50)	0.10	

	Chlorpyrifos	OP	> 56 LD^b^	2.15 (0.96–4.81)	0.36	Lee et al. 2004a
			> 417 IWLD^b^	3.01 (1.35–6.69)	0.15	

	Diazinon	OP	> 39 LD^c^	3.36 (1.08–10.5)	0.026	Beane Freeman et al. 2005
			Highest IWLD^c^	2.88 (0.92–9.03)	0.053	

	EPTC	Thiocarbamate	> 50 LD^c^	2.36 (1.16–4.84)	0.02	van Bemmel et al. 2008
			> 112 IWLD^c^	1.87 (0.97–3.59)	0.05	

	Fonofos	OP	> 609 IWLD^c^	2.67 (1.06–6.70)	0.04	Mahajan et al. 2006a

All LH	Alachlor	Chloroacetanilide	> 116 LD^c^	2.04 (0.89–4.65)	0.02	Lee et al. 2004b
			> 710 IWLD^c^	2.42 (1.00–5.89)	0.03	

	Chlorpyrifos	OP	> 56 LD^b^	1.43 (0.86–2.36)	0.26	Lee et al. 2004a
			> 417 IWLD^b^	1.99 (1.22–3.26)	0.09	

	Diazinon	OP	> 39 LD^c^	1.84 (0.89–3.82)	0.094	Beane Freeman et al. 2005
			Highest IWLD^c^	2.01 (1.02–3.94)	0.049	

	Permethrin	Pyrethroid	> 50 LD^c^	1.64 (1.07–2.52)	0.35	Rusiecki et al. 2009
			> 220 IWLD^c^	1.31 (0.84–2.04)	0.60	

NHL	Lindane	OC	> 22 LD^d^	2.10 (0.80–5.50)	0.12	Purdue et al. 2006
			Highest IWLD^d^	2.60 (1.10–6.40)	0.04	

Multiple myeloma	Permethrin	Pyrethroid	> 50 LD^c^	5.72 (2.76–11.8)	< 0.01	Rusiecki et al. 2009
			> 220 IWLD^c^	5.01 (2.41–10.4)	< 0.01	

Bladder	Imazethapyr	Imidazolinone	> 311 IWLD^b^	2.37 (1.20–4.68)	0.01	Koutros et al. 2009

Prostate	Fonofos	OP	> 56 LD^c^	1.77 (1.03–3.05)	0.02	Mahajan et al. 2006a (for applicators with a family history of prostate cancer)
			> 315 IWLD^c^	1.83 (1.12–3.00)	0.01	

	Methylbromide	Halogenated alkane	Highest IWLD^e^	3.47 (1.37–8.76)	0.004	Alavanja et al. 2003

Brain	Chlorpyrifos	OP	> 56 LD^b^	2.58 (0.73–9.17)	0.076	Lee et al. 2004a
			> 417 IWLD^b^	4.03 (1.18–13.8)	0.036	

Melanoma	Carbaryl	Carbamate	> 175 LD^b^	4.11 (1.33–12.7)	0.07	Mahajan et al. 2007
			Highest intensity score^b^	1.54 (0.61–3.86)	0.92	

	Toxaphene	OC	> 25 LD^d^	2.90 (1.10–8.10)	0.03	Purdue et al. 2006
			Highest IWLD^d^	1.80 (0.70–5.10)	0.24	

Abbreviations: LH, lymphohematopoietic cancers; OC, organochlorine; OP, organophosphate. ORs were reported by Alavanja et al. (2003, 2004), Andreotti et al. (2009), and Lee et al. (2007b); all others are RRs.

a All RRs and ORs were estimated relative to nonexposed applicators except those reported for alachlor and all LH (Lee et al. 2004b) and dicamba and colon (Samanic et al. 2006) and lung cancer (Alavanja et al. 2004), which are in reference to applicators in the lowest category of exposure.

b Highest quintile.

c Highest quartile.

d Highest tertile.

e Highest sixth.

**Table 2:** Evidence of carcinogenicity noted as of March 2009 by the U.S. EPA, PMRA, and IARC for registered pesticides that displayed a significant exposure–response relationship with at least one type of cancer

Pesticide	Type	Cancer type(s) with exposure–response in the AHS cohort	Organization		
			U.S. EPA	PMRA	IARC
Alachlor (Lee et al. 2004b)	Herbicide	All LH	Likely (high doses)/not likely (low doses)	Not registered in Canada	Not evaluated
Aldicarb (Lee et al. 2007a)	Insecticide	Colon	Group E^a^	Not registered in Canada	Group 3^b^
Carbaryl (Mahajan et al. 2007)	Insecticide	Melanoma	Likely	Under re-evaluation (positive)^c^	Group 3^b^
Chlorpyrifos (Lee et al. 2004a, 2007b)	Insecticide	Lung, rectum	Group E^a^	Negative^d^	Not evaluated
Diazinon (Alavanja et al. 2004; Beane Freeman et al. 2005)	Insecticide	All cancers, all LH, leukemia, lung	Not likely	Negative^d^	Not evaluated
Dicamba (Alavanja et al. 2004; Samanic et al. 2006)	Herbicide	Colon, lung	Not likely	Negative^d^	Not evaluated
EPTC (Andreotti et al. 2009; van Bemmel et al. 2008)	Herbicide	All cancers, colon, leukemia, pancreas	Not likely	Negative^d^	Not evaluated
Imazethapyr (Koutros et al. 2009)	Herbicide	Bladder, colon	Not likely	Under re-evaluation (negative)^d^	Not evaluated
Metolachlor (Alavanja et al. 2004)	Herbicide	Lung	Group C^e^	Not registered in Canada	Not evaluated
Pendimethalin (Alavanja et al. 2004; Andreotti et al. 2009; Hou et al. 2006)	Herbicide	Lung, rectum, pancreas	Group C^e^	Positive^c^	Not evaluated
Permethrin (Rusiecki et al. 2006, 2009)	Insecticide	Myeloma	Likely	Positive^c^	Group 3^b^
Trifluralin (Kang et al. 2008)	Herbicide	Colon	Group C^e^	Positive^c^	Not evaluated

a Evidence of noncarcinogenicity in humans.

b Not classifiable as to carcinogenicity to humans.

c Evidence of carcinogenicity noted in animal toxicology database.

d No evidence of carcinogenicity in animal toxicology database.

e Possible human carcinogen.
